# Happy children! A network of psychological and environmental factors associated with the development of positive affect in 9–13 children

**DOI:** 10.1371/journal.pone.0307560

**Published:** 2024-09-06

**Authors:** Tommaso Feraco, Giorgia Cona

**Affiliations:** 1 Department of General Psychology, University of Padova, Padua, Italy; 2 Department of Neuroscience, University of Padova, Padua, Italy; 3 Padua Neuroscience Center, University of Padova, Padua, Italy; Private University Schloss Seeburg: Privatuniversitat Schloss Seeburg, AUSTRIA

## Abstract

To deepen the development of positive affect during early adolescence and shed new light on its predictors, this study adopts an exploratory network approach to first identify the main domains that describe the variability of children’s psychological, environmental, and behavioral characteristics, and then use these domains to longitudinally predict positive affect and its development within a latent growth framework. To this aim, we considered 10,904 US participants (9 years old at baseline; 13 years old 42 months later), six measurement occasions of positive affect, and 46 baseline indicators from the ABCD study. Our results not only confirm that positive affect declines between 9 and 13 years old, but also show that among the five domains identified (behavioral dysregulation, cognitive functioning, psychological problems, supportive social environment, and extracurricular activities), only a supportive social environment consistently predicts positive affect. This is crucial for practitioners and policymakers, as it can help them focus on the elements within our complex network of psychological, social, and environmental variability.

## Introduction

*«Viuere, Gallio frater, omnes beate uolunt, sed ad peruidendum quid sit quod beatam uitam efficiat caligant»*

*«Everyone, brother Gallione, wishes to live happily, but is dull at perceiving exactly what it is that makes life happy»*

De Vita Beata ("On the Happy Life", 58 AD)

Around the year 58 AD, the Latin philosopher Seneca wrote ‘De Vita Beata’ (On the Happy Life), a dialogue with his brother Gallio, in which he defined the concept of the happy life and discussed how it can be achieved. Interestingly, long time after, in the pandemic year 2020, the term ‘happiness’ was shown to be searched more than ever on Google Search. These two, extremely far, examples underline an ancestral human need. Indeed, identifying what are the key factors to pursuit happiness has represented and still represents a central question for humans.

The study of happiness, as well as the strictly related terms ‘subjective well-being’ and ‘life satisfaction’, has grown in popularity within the discipline of psychology [1], with developmental psychologists being particularly interested in its variability and decline in early adolescence [2], which is classically considered a challenging period. Indeed, adolescence represents a critical period in which children face substantial changes in almost every aspect of their lives. In particular, during the transition from childhood to adulthood, adolescents encounter significant hormonal changes that influence their physical, sexual, and brain maturation and develop cognitive abilities and more stable personality traits [3–6]. Changes, however, also encompass their social lives: adolescents are required to make choices for their own future, take responsibilities and achieve increasing independence. They also change schools and schoolmates, friendships, build their first intimate relationships, and actively engage in extracurricular activities and with the larger community [7–10]. All these changes might lead to a psychological crisis for adolescents, who might encounter emotional and health problems, including depression and mental disorders, and might represent risk factors for future substance use and abuse [7, 11]. Indeed, adolescence has been associated with the steepest decline in subjective well-being of the entire life span [10, 12]. Some researchers argue that this is because children have not yet developed the necessary abilities to control and regulate their emotions, which are consequently disrupted by the new challenges of this transition phase [9, 13, 14]. However, having high positive emotions remains crucial for a positive and healthy development. Indeed, positive affect longitudinally predicts mental and physical health, effective coping, positive social relationships, and success in adolescence and across the entire lifespan [15–19]. Positive affect, for example, is associated with improved cardiovascular (e.g., lower heart rate and blood pressure) neuroendocrine (e.g., cortisol levels) and immune systems [17, 20, 21]. Additionally, positive affect leads to healthy outcomes through social and behavioral pathways as it reduces risky behaviors, while increasing social support, friends and positive relationships, which are all predictors of better mental health [19–21].

Given the centrality of positive emotions and the detrimental effects that low positive affect could cause, it is fundamental to identify the patterns of development and the main predictors of positive affect in the first years of adolescence to understand how it might be possible to favor a successful and positive development in adolescence. To answer these questions we take advantage of the longitudinal Adolescent Brain Cognitive Development data collection (ABCD; Volkow et al., 2018; https://nda.nih.gov/abcd), a large database of data collected in the United States (US).

### Positive affect in adolescence

Despite a common definition of emotions and affect is yet to be found, affect can be described as a general experience of feelings or emotions that are distributed along two dimensions of valence and intensity [22]. Specifically, we refer to positive affect when the person feels pleasant or positive emotions (e.g., joy, calm, interest, pride); contrarily, we refer to negative affect when the person feels negative emotions like stress, fear, or anger. Importantly, affect encompasses a generally stable tendency of the individual toward experiencing more or less positive and negative emotions [23]. Studies interested in developmental affective changes focused on (a) the variability of affective experiences and (b) the age-related trajectories of mean level scores in positive and negative affect. Maciejewski and colleagues [14, 24], for example, in a 5-year longitudinal diary study report that affective variability decreases in late adolescence (i.e., negative and positive affect are more stable in late adolescence) and that the mean level scores in positive affect decreases, while negative affect increases from early to middle adolescence. In a larger study, Griffith and colleagues [22] confirmed these findings on a sample of 9–17 years old participants who were asked to report positive and negative affect every 18 months for three years. In particular, the authors found that positive affect linearly decreases with age while negative affect follows a curvilinear pattern in the total sample. These results are also in line with changes in personality and emotion regulation strategies [25–28] that show an increase in neuroticism and extraversion and a decrease in emotion regulation strategies during early adolescence. In other words, it is expected that positive affect decreases in early and middle adolescence, but more research is needed to corroborate these findings as also the authors [22] highlight that they contradict previous studies on children between 9 and 13 years old which show an increase in positive affect during this early period [29]. The ABCD data collection, which includes an extremely large amount of longitudinal data, could be precious to better understand how positive affect develops between 9 and 18 years old. Importantly, the ABCD study includes a plethora of additional information giving the opportunity to also identify the predictors of positive affect, at least in US children. The ABCD team has already released data from 9 to 13 years old, allowing the study of the critical period characterizing the end of childhood and the first years of adolescence.

### What makes a child happier? Correlates of positive emotions in adolescence

While the study of affective development is fundamental to understand the age of adolescence, a further and crucial step is to detect those factors that possibly promote higher levels of positive affect during adolescence [27]. Other than neural and biophysiological characteristics [10, 30, 31], there are many social, environmental, and psychological aspects that may concur to higher or lower levels of positive and negative affect. One key factor are interpersonal relationships with peers, parents, and teachers [32–34], but other studies and meta-analyses highlight the role of many other factors in explaining and supporting positive affect in adolescence. Among the many, there are physical activity [35], sleep [36], personality traits [37], academic achievement [38], nature connectedness and environment [39, 40], the practice of leisure activities [41, 42], psychological and behavioural problems [43, 44], or social networks and use of phones, tablets, and computers [45, 46].

#### Toward a low dimensionality of the predictors of positive affect

This plethora of results and meta-analyses on the topic highlight the attention that the study of positive affect’s correlates has received in recent years. For practical reasons, however, most studies focus on a narrow group of specific variables and cannot take in consideration the vast majority of the other factors that might be linked to positive affect. Nonetheless, most psychological variables are correlated between each other and pondering on or taking into account such multiple and mutual associations is an important key to better understand psychological phenomena. Indeed, it is an emerging matter of discussion that the psychological realm of individuals could be better represented by a network of superordinate domains [47–53]. In other words, if we believe that psychological dimensions are not independent but instead interact with each other and with various characteristics of the individual and their environment, then bivariate associations poorly describe their interrelationships. It is therefore needed to adopt alternative approaches to unveil the pattern among a wide set of inter-related variables, such as network analysis.

In addition, recent strands of research are accompanying the reflection on psychological networks with the concept of low dimensionality. In fact, network analyses highlight regions (domains) of highly interconnected nodes (variables) that work together following specific patterns of associations and that are less related with nodes from other domains. The researchers from different fields are increasingly focusing on hierarchical models of personality, character, or psychopathology [54–60] rather than considering the multiplicity of specific factors or abilities as separate and isolated variables. Smith et al. (2015), for example, found that people spread along a single positive-negative continuum linking brain connectivity, lifestyle, intelligence, and demographic variables. This suggests that a single mode of population variation can explain most of the variance in distinct psychological aspects. Moreover, Granziol and Cona (2023) recently found that the 38 variables included in their large study are well described by a network of seven separate but inter-related domains. Such low dimensionality of psychological data could help to highlight which are the main—hidden—domains that characterize social, environmental, and psychological data and to understand which—among the few instead of dozens of interrelated variables, should be the focus of research and interventions. In the present context, this would be translated in identifying the core psychological and/or environmental dimensions that can promote high positive affect.

Despite the potentialities of this approach are enormous, gathering this kind of data is extremely difficult because individuals should be measured on a large spectrum of variables and a large population is necessary to obtain precise estimates of the models/networks. Fortunately, large and open data collection projects are on the rise and could serve to effectively study the best predictors of positive affect across a large spectrum of psychological, environmental, and social data. Again, the ABCD perfectly fits this aim and also gives the opportunity to study such association longitudinally and in youths. The project, in fact, aims to longitudinally collect a large amount of data from more than 10,000 US children aged 9–10 for a period of ten years. Importantly, they already measured children’s positive affect at six different time points, as well as a plenty of other features (e.g., cognitive, environmental, psychological features). This allowed us to establish a network of their psychological domains at the baseline and understand which of the domains individuated best predict positive affect and its development over 42 months.

### Aims of the study

Taking advantage of the large and longitudinal ABCD data collection, this study aimed to answer two main research questions regarding positive affect:

- How does positive affect develop between 9 and 13 years old? To this aim, measures of positive affect from 7 time points were analyzed using latent growth models.
- What are the main predictors of positive affect and its development between 9 and 13 years old? To this aim 46 variables generally concerning mental health, physical health, environment, and neurocognition were considered as possible predictors of positive affect and its development. To decrease the dimensionality of the predictors, we decided to follow a network approach [61] to firstly analyse the network structure of the ABCD variables and use the domains individuated to longitudinally predict (across six waves and up to three years) children’s positive affect.

Our work could be of primary importance as it will (a) accumulate knowledge on the topic of positive affect development, (b) inform us about the network structure (even if always inevitably partial) of youths’ variability and, importantly, (c) show which are the more relevant domains in longitudinally predicting a fundamental developmental factor such as positive affect. In addition, the network approach and the focus on “umbrella domains”–instead of inspecting multiple single variables—follow recent evidence sustaining a network structure of various psychological domains underpinning a lower dimensionality of psychological variables [48, 49, 62].

## Materials and methods

### Participants

To the aim of this study, we used archive data provided by the ABCD project (https://nda.nih.gov/abcd). The ABCD is a prospective longitudinal study starting with children aged 9–10 that will be followed for ten years [63]. Exclusion criteria were limited to proficiency in English language, severe sensory, intellectual, medical, or neurological issues, and contraindications to MRI scanning. We accessed the data on December 18^th^ 2022. No information that could identify individual participants was provided.

A total of 11,878 children were initially enrolled at 21 research sites across the USA and evaluated on different aspects including mental health, environmental factors, substance use and attitudes, biospecimens, physical health and sport involvement, culture, cognitive abilities, and social and emotional development. fMRI was also used to measure brain development and its correlates through adolescence. ABCD data are released every year and we took advantage of the last data release available at the time of writing: Data Release 4.0 (https://nda.nih.gov/study.html?id=1299). Other than imaging data that will not be included in our analysis, this release contains non-imaging assessment at baseline and follow-up for seven different time points: baseline, 6 months, 12 months, 18 months (full cohort), 30 months, 36 months, and 42 months (interim data).

We specifically included all participants who showed no missing values on the selected variables (see below) at the baseline. Longitudinally, we only included information about positive affects at the different data collection points. The full baseline sample consisted of 10904 children (Females = 5226) aged 9.91 in mean (SD = .62). The numerosity differed at the subsequent time points depending on how many children that filled the positive affect measure were made available in the Data Release 4.0 (see Table 1). A total of 2629 participants always completed the survey and had no missing values in any variable, including demographics. As stated before, the last three waves of data are interim data and missing data do not correspond to participants’ dropout, but they are mostly data that still must be processed and made available by the ABCD team.

**Table 1:** Numerosity at each time point. Females are reported within parenthesis.

	Baseline	6 months	12 months	18 months	30 months	36 months	42 months	Complete
N (Females)	10904 (5226)	9621 (4598)	9466 (4532)	9392 (4482)	7249 (3479)	5280 (2491)	2816 (1356)	2629 (1272)
M age (SD)	9.92 (0.63)	9.92 (0.63)	9.91 (0.63)	9.91 (0.63)	9.93 (0.62)	9.98 (0.62)	10.01 (0.6)	10.01 (0.6)

*Note*. Age always refers to participants’ age at the baseline

### Materials

Assessment measures were broadly included into nine different main areas by the ABCD team: substance use, mental health, physical health, culture, environment, neurocognition, genetics, mobile technology, and imaging data. To the aim of our study, we selected only mental health, physical health, environment, and neurocognition areas (see S1 Table). Indeed, substance use variables were not informative at the baseline as almost no children reported using any substance at this age; mobile technology measures were not collected at the baseline; and imaging and genetics data were not the focus of our work. In case the same measure was filled twice, once by the children and once by the parent, we decided to consider the children version (e.g., prosocial behaviour, screen time, family conflict). Given the focus of our study on children characteristics, we also excluded measures directly referring to parents and filled by the parents (e.g., measures of parents’ substance use or psychopathology). Measures of brain injury, culture (e.g., black, Hispanic, American Indian), medication use, substance use, and personal information such as handedness, vision, or weight were not used. Indeed, specific medical information are beyond the scope of our study and may have medical and biological implication that are not in our field of expertise.

A summary of the measures included in this study is available in S1 Table.

#### Demographic measures

A demographic questionnaire adapted from the PhenX toolkit [64] was used to collect information about the children and their parents/guardians [65]. These included sex at birth, age, race, ethnicity, family income, and parent’s education. Only information about the responding parent were used because information about the second partner was often missing without specification of the reason. Sex, age, family income, and parent’s education will be entered as covariates in the analyses.

#### Mental health measures

A series of questionnaires were used to measure mental health factors. The abbreviated youth version of the UPPS-P Impulsive Behavior Scale [66] was used to measure five facets of impulsivity behaviour: lack of perseverance, lack of planning, sensation seeking, negative urgency, and positive urgency. The abbreviated Prodromal Psychosis Scale [67] was used as index of subclinical psychosis risk phenotypes. The BIS/BAS scale [68] was used to measure three facets of behavioural activation reflecting positive affect: Fun Seeking, Drive, and Reward Responsiveness, and Behavioural Inhibition scale. The number of friends and close friends was also measured with ad-hoc questions (i.e., Friendship scale). The Parent General Behavior Inventory [69] was used to measure symptoms of mania. The total score of the Achenbach Child Behavior Check List [70] was used as a measure of children’s internalizing and externalizing behavioural problems. The computerized version of the Kiddie-Structured Assessment for Affective Disorders and Schizophrenia for DSM-5 [65, 71] was used as a diagnostic interview measuring depression, anxiety disorders, somatic disorder, ADHD, oppositional conductive disorders, conduct disorders, obsessive compulsive disorders, sluggish cognitive tempo, and stress disorders. Raw scores were used for each of these.

A complete description of the measures included in the Mental Health area is available in the corresponding ABCD study [65].

#### Physical health measures

The physical health area included measures of physical activity, in terms of amount of weekly physical activity [72]; screen time, as a sum of time spent watching TV, videos, or playing video games, chatting, and using social network during a typical day [73, 74]; involvement in sport and other activities, as a measure of months in which the children practiced a sport or a hobby during their past life [75]; and sleep disturbance [76].

A complete description of the measures included in the Physical Health area is available in the corresponding ABCD study [65].

#### Culture and environment measures

The culture and environment area included measures of prosocial behaviour [77]; parental monitoring, or parent’s active efforts to keep track of child’s whereabouts [78]; parent’s acceptance reported by the children [79, 80]; family conflicts, or the amount of openly expressed conflicts among family members [81]; three subscales of the School Risk and Protective Factors Survey [82] were used to measure school environment, school involvement, and school disengagement. Information about children’s neighbourhood, in terms of feelings about safety and presence of crime in the respondent’s neighbourhood, were obtained using the ABCD Youth Neighbourhood Safety/Crime Survey Modified from PhenX [83]. Information about culture (e.g., spoken language, family heritage) were not included in our analysis.

A complete description of the measures included in the Culture and Environment area is available in the corresponding ABCD publication [84].

#### Neurocognition measures

A 70-minute-long neurocognitive battery including ten different measures was administered with an iPad. These measures included seven tasks from the NIH Toolbox^®^ (http://www.nihtoolbox.org) evaluating episodic memory (Picture Sequence Memory Test), inhibition and conflict monitoring (Flanker Task), cognitive flexibility (Dimensional Change Card Sort Task), working memory (List Sorting Working Memory Test), processing speed (Pattern Comparison Processing Speed Test), verbal abilities (Picture Vocabulary Task), reading (Oral Reading Recognition Task). Additionally, the Rey Auditory Verbal Learning Test was used to measure learning, memory and recognition [85]; the Little Man Task to measure visuospatial abilities [86]; and the Matrix Reasoning Task from the Wechsler Intelligence Test for Children-V [87] to measure fluid intelligence. A single item measure of delay of gratification with dichotomous response [88] was also included in the neurocognition tasks, but it will not be included in the analysis because it does not load on cognitive factors [89].

A complete description of the neurocognitive battery is available in the ABCD publication by Luciana and collaborators [88].

#### Positive affect

The Positive Affect Scale from the NIH Toolbox Battery [90] was administered at different time points but not at the baseline (i.e., after 6, 12, 18, 30, 36, and 42 months from the baseline) to evaluate positive emotions and affective well-being in past week [90]. Positive affect has been characterized as “feelings that reflect a level of pleasurable engagement with the environment such as happiness, joy, excitement, enthusiasm, and contentment (p. 4, Salsman et al., 2013). Respondents were provided with 9 positive affects items (e.g., “I felt calm”, “I felt delighted”) and indicated on a 3-points Likert scale (1 = Not true; 3 = Very true) how much they felt that way in the last week. The scale showed high internal validity (Cronbach’s alpha = .96).

## Statistical analysis

To the aims of the study, two main groups of analyses were run. The first to detect what are the main social, environmental, and psychological domains of the baseline network structure of ABCD variables and the second to study the development of positive affect and its association with the domains individuated (i.e., what makes children happy?). All the analysis were run in R [91].

Given the large number of participants included in the study, we decided not to rely on significance of the effects (which are more likely to result significant) but only on the magnitude of the effects. In particular, we will interpret only effects with an associated standardized effect of.10 or higher as practically useful in line with the principles of the region of practical equivalence [92, 93].

### Exploratory graph analysis

**Fig 1:**
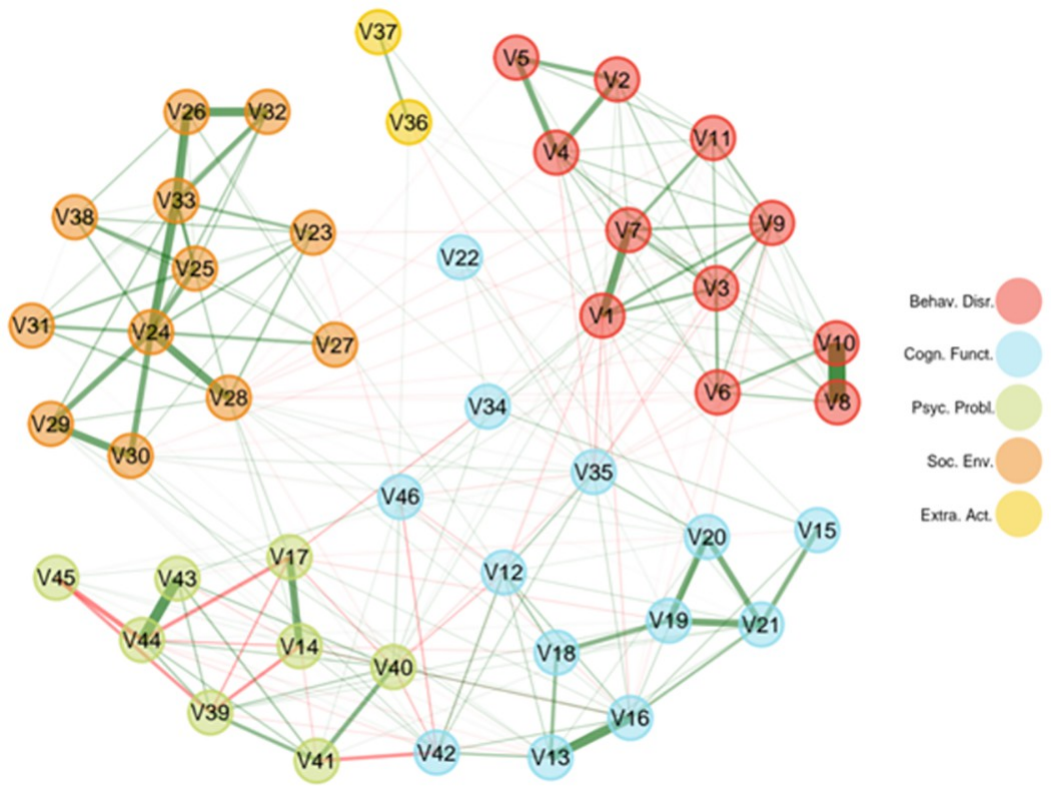
Graphical representation of the network. Each colored circle represents a variable and its domain (1 to 5). Lines indicate associations between variables: green lines for positive relations and red lines for negative relations. The width of the lines represents the strength of the association. **Note.** V1 = vocabulary; V2 = inhibition; V3 = working memory; V4 = cognitive flexibility; V5 = processing speed; V6 = episodic memory; V7 = reading; V8 = verbal memory; V9 = fluid reasoning; V10 = long term memory; V11 = visuospatial abilities; V12 = prodromal psychosis; V13 = negative urgency; V14 = lack of planning; V15 = sensation seeking; V16 = positive urgency; V17 = Lack of perseverance; V18 = behavioral inhibition (bis); V19 = reward responsiveness (bas); V20 = drive (bas); V21 = fun seeking (bas); V22 = friends; V23 = symptoms of mania; V24 = total ext-int problems; V25 = depression; V26 = anxiety disorders; V27 = somatic disorder; V28 = ADHD; V29 = oppositional conductive disorders; V30 = conduct disorders; V31 = sluggish cognitive tempo; V32 = obsessive compulsive disorders; V33 = stress disorders; V34 = weekly physical activity; V35 = screen time; V36 = sport activity; V37 = hobbies/other activities; V38 = sleep disturbance; V39 = prosocial behaviour; V40 = parental monitoring; V41 = parent’s acceptance; V42 = family conflict; V43 = school environment; V44 = school involvement; V45 = school disengagement; V46 = safe neighbourhood.

An Exploratory Graph Analysis (EGA) approach was adopted to study the underlying network of connections between the measures included in our study. EGA is a recently proposed technique [61] that, starting from the matrix of partial correlations between a set of variables, allows to estimate the number of domains to be retained and also estimates and graphically represents the associations (i.e., edges) between the measures (i.e., nodes) [94]. After the network is estimated, EGA provides different indices including the strength of the association between each node and its dimension, which is equivalent to factor loadings in factor analysis [95] and the strength of the edges, which represents the semipartial correlation between two nodes and is represented in the network graph (see Fig 1) with thinner or thicker lines depending on the magnitude of the association [50]. EGA has different advantages and an equal or better performance than other traditional measures (i.e., VSS, MAP, K1, BIC, EBIC, PA). A first advantage is that it relies less on the subjectivity of the researcher, who must traditionally choose the rotation of the loadings matrix [96]; it also allows for a graphical and easy to interpret representation of the model (see Fig 1); and has the practical advantage of estimating the number of dimensions, the edges, and the loadings within the same algorithm, without using two different set of analyses. For what concerns actual statistical performance, a recent simulation study showed that EGA has high accuracy in all the condition tested (varying number of factors, sample size, factor loadings, number of variables, factor correlations, and number of response options) and that it has the best accuracy to correctly estimate the number of factors and the lowest mean bias error [94] across condition. Parallel analysis (PA) also performed very well across most conditions, but EGA outperformed it in large datasets and large structures (e.g., four or more domains), as in our case [61, 94]. For these reasons, EGA is perfectly suited to the aims of our study.

To ensure that a network is reliable, stable, and not sample-specific, it is possible to follow a bootstrap procedure [97]. In this case we applied a non-parametric bootstrap with 10,000 replications. The median network obtained via bootstrap was then compared with the original network and the stability of the network was calculated as the proportion of times the network was equally replicated in the bootstraps. Additionally, we inspected the reliability of each node looking at how many times the same node belonged to each domain of the network. A node is assumed to be stable when it appears at least 80% of the times as loading on the same component [97]. The EGAnet package was used for this analysis [98]. Finally, the *net*.*scores()* function was used to extract participants’ latent scores for each dimension of the estimated network. These scores will serve as predictors of positive affect in subsequent analysis.

### The development of positive affect and its association with the ABCD dimensions

To study the development of positive affect and its associations with the domains extracted following the EGA, we used a latent growth model. Factor loadings of the observed composite variables were fixed at 1 for each time point. The loadings for the linear change factor were fixed in ascending order as 0 (6 months), 1 (12 months), 2 (18 months), 4 (30 months), 5 (36 months), and 6 (42 months; 3 is missing because participants were not contacted after 24 months. Predictors were the scores extracted from the network. *z scores* in positive affect were used after scaling positive affect on the whole sample. To handle missing data and avoid losing information we used full information maximum likelihood (FIML) to estimate missing data. Goodness-of-fit was evaluated using the Comparative Fit Index (CFI), the Goodness of Fit Index (GFI), the Standardized Root Mean Squared Residual (SRMR), and the Root Mean Square Error of Aproximation (RMSEA) [99].

The R packages Lavaan [100] was used for this set of analyses.

## Results

### Exploratory graph analysis

The EGA run on the baseline sample including 10904 participants, shows that the 46 variables included in this analysis can be aptly described by five inter-related domains (see Fig 1) that we interpreted and named as behavioural dysregulation (D1), cognitive functioning (D2), psychological problems (D3), supportive social environment (D4), and extracurricular activities (D5). The behavioural dysregulation domain is composed by 13 nodes (variables) including the four BIS/BAS measures, physical activity, screen time, the number of friends, safe neighbourhoods, sensation seeking, negative and positive urgency (UPPS scale), family conflict, and prodromal psychosis. This domain was labelled as behavioural dysregulation because the most central and correlated nodes of the domain encompass behavioural inhibition and activation systems, negative and positive urgency, and sensation seeking. The other nodes were less associated, but might be proxies of behavioural dysregulation (e.g., individuals with higher behavioural dysregulation might encounter higher family conflicts). The cognitive functioning domain included all the 11 measures of neurocognition. The psychological problems domain was composed by 12 nodes including behavioural problems, sleep disturbance, mania symptoms, and the disorders measured with the K-SADS interview (i.e., conduct, oppositive, somatic, stress, ADHD, sluggish cognitive tempo, obsessive compulsive disorder, anxiety, and depression). The supportive social environment domain is composed of 8 nodes including the three measures of school environment, prosocial behaviour, parent acceptance and monitoring, and the two measures of the UPPS scale for lack of perseverance and lack of planning. Finally, the extracurricular activities domain included two nodes measuring the months spent by the children doing sport or other activities (e.g., hobbies). All the standardized loadings of the network are available in S2 Table.

Finally, the bootstrap procedure suggests that the network individuated is highly stable. Specifically, it replicated exactly 99% of the times. Nodes were also highly stable and none of them was under the.80 cut-off. Physical activity (V34, behavioural dysregulation) was the less stable node with a replication of 94%. It appeared in the fourth domain on 6% of the occasions. The complete stability results are pictured in S1 Fig.

Given the goodness of the model, latent domain scores for the five domains were extracted and used in the following analyses. Pearson correlations between domain scores and the other variables are available in Table 2. The five factors were only slightly correlated between each other.

**Table 2:** Pearson correlations between the five dimensions, positive affect at different time points, and covariates. Correlations ≥ |.10| are reported in bold.

	1.	2.	3.	4.	5.	6.	7.	8.	9.	10.	11.	12.	13.	14.
1. D1	-													
2. D2	**-.34**	-												
3. D3	**.25**	**-.18**	-											
4. D4	**-.25**	**.14**	**-.22**	-										
5. D5	**-.22**	**.38**	-.07	**.13**	-									
6. PA 6	-.09	**.16**	**-.10**	**.28**	**.12**	-								
7. PA 12	-.03	.07	-.09	**.30**	.06	**.35**	-							
8. PA 18	-.12	**.15**	**-.14**	**.28**	**.11**	**.40**	**.41**	-						
9. PA 30	**-.10**	**.12**	**-.15**	**.24**	.09	**.30**	**.34**	**.43**	**-**					
10. PA 36	-.06	.04	**-.11**	**.25**	.02	**.25**	**.36**	**.39**	**.50**	-				
11. PA 42	-.06	.05	**-.14**	**.20**	.05	**.24**	**.28**	**.36**	**.49**	**.52**	-			
12. Age	-.07	**.27**	-.01	.04	**.13**	.02	.00	-.02	-.04	-.01	-.03	-		
13. Sex (M)	**-.13**	.05	**-.10**	**.20**	**.10**	.08	.04	.06	.00	-.04	-.07	-.02	-	
14. Income	**-.25**	**.44**	**-.17**	.06	**.32**	**.10**	.04	**.12**	**.10**	.06	.04	.04	-.00	-
15. Education	**-.20**	**.40**	-.09	.04	**.31**	**.11**	.03	**.13**	**.11**	.05	.05	.02	-.01	**.62**

*Note*. PA = Positive Affect; D1 = behavioural dysregulation; D2 = cognitive functioning; D3 = psychological problems; D4 = supportive social environment; D5 = extracurricular activities

### The development of positive affect and its relationship with the ABCD dimensions

Results of the latent growth model show that the model adequately fit the data as indicated by three of the four indices adopted (SRMR = .04; RMESA = .06; GFI = .95). The CFI (.89) was slightly lower than what is deemed acceptable. The latent intercept value for positive affect was slightly higher than the mean (*β*_intercept_ = .23; *b* = .14) with a latent slope value indicating that positive affect decreases with time (*β*_slope_ = -.48; *b* = -.06). Intercept and slope resulted slightly correlated (*r* = -.25) indicating that the higher is positive affect at the baseline, the steepest is the decline. The effect of the predictors on the latent intercept was the biggest for *supportive social environment (β =* .*43*; *b* = .27*)*. Psychological problems showed a very small association with positive affect intercept (*β* = .15; *b* = .09). Behavioural dysregulation (*β* = .06; *b* = .04), cognitive functioning (*β* = -.04; *b* = -.02), and extracurricular activities (*β* = .08; *b* = .05) showed negligible effects on the intercept. The slope was almost unaffected by the predictors with only cognitive functioning and psychological problems having a small but almost negligible association with positive affect’s slope (*β* = -.11; *b* = -.01 in both cases). The effect of behavioural dysregulation (*β* = -.05; *b* = -.01), supportive social environment (*β* = -.08; *b* = -.01), and extracurricular activities (*β* = -.05; *b* = -.01) on the slope was practically negligible. Betas (*β*) indicate standardized coefficients while *b*s (*b*) indicate unstandardized coefficients. This model explained 25% of the intercept variance, and 3% of the slope variance.

## Discussion and conclusions

Adolescence is a period of changes and adolescents face social insecurity, uncertainty, and higher responsibilities. Being able to functionally adapt to such requests is a hard job that may undermine one’s well-being and positive affect, as evident from their decline highlighted in early adolescence [12, 22]. A decline in positive affect, however, may lead to additional difficulties in areas that include physical and mental health, cognitive functioning, school success, sociality, and decision making, among the others [15–20, 101]. For this reason, it is crucial to further investigate the development of positive affect and its antecedents.

In the light of these observations, our study had two main aims: the first, and preliminary, aim was to study the network of associations among a large number of variables pertaining to social, environmental, and psychological characteristics of the children; the second was to study the longitudinal development of positive affect between 9 and 13 years old and its association between the domains individuated in the network. The first aim, in line with recent studies suggesting that different psychological facets can be well described by a lower number of network domains [48, 49, 62], allowed us to reduce the number of possible variables to be considered as predictors of positive affect while taking into account their entire network of associations and their latent superordinate structure. This could help overcoming the problems inherent to previous research in the field (and in psychology in general) in which narrow predictors have been used, causing a proliferation of studies and meta-analyses that do not account for the complex structure of psychological, social, and environmental variables. Contrarily, with a network approach, educators, policymakers, institutions, or whoever is involved in promoting positive affect, could focus on a restricted range of aspects that might be more effective to the goal. In fact, out of the five domains individuated and—by literature—plausibly associable to positive affect, only one resulted predicting it strongly in children: a supportive social environment.

### The network structure

Recent psychological literature [48, 49, 52, 62] highlights the tendency toward a lower dimensionality of psychological constructs. For practical reasons, however, most studies narrow their focus to specific variables and do not consider the high interconnection that binds psychological, social, and environmental variables. A large study that identifies the network of associations between a large number of variables could thus help to understand their reciprocal association and also to reduce the number of domains that we should focus on to promote positive affect in adolescence.

The EGA applied on the 46 baseline variables included has individuated five stable and interconnected domains in the network structure: behavioural dysregulation, cognitive functioning, psychological problems, supportive social environment, and extracurricular activities. The ‘behavioural dysregulation’ domain encompasses activities and attitudes characterized by high energy, impulsive behaviours, and the need for new experiences and people. Surprisingly, it also includes family conflicts and safe neighbourhoods. Indeed, while one might expect these nodes as belonging to the ‘supportive social environment’ domain, they instead represent a bridge between ‘behavioural dysregulation’ and ‘supportive social environment’ (see Fig 1) possibly connecting the tendency to develop a less regulated behaviour to the presence of a less supportive social environment. This is in line with previous studies highlighting that the social environment could drive on the long term to deviant, impulsive, and uncontrolled behaviours [84, 102–104]. In other words, the way one behaves and their social environment are two separate but entangled dimensions. The ‘supportive social environment’ domain, on the other side, is characterized by variables concerning a positive adaptation or belongingness to the most influent social environments to which children are exposed: school and their family. Together with these aspects, a supportive environment seems to favour children’s future planning abilities in the sense that such children are more projected into the future and tend to persevere and plan their future more than others. This is highlighted by the two future-oriented subscales of the UPPS scale (i.e., lack of perseverance and lack of planning) that emerged as part of this domain and not of the first domain (i.e., ‘behavioural dysregulation’ domain) wherein the present-oriented subscales were included (i.e., sensation seeking, and positive and negative urgency). The ‘cognitive functioning’ domain clearly reflects the well-known *g factor* [105] of intelligence and separates it from the other not strictly cognitive domains, highlighting a distance between cognitive variables and social, psychopathological, environmental, and behavioural nodes. The ‘psychological problems’ domain is composed of all—with the exception of prodromal psychosis symptoms—the externalizing and internalizing problems measured. This is perfectly in line with new evidence in literature suggesting the existence of an overarching psychopathology factor [57, 106, 107], as already emerged in other ABCD studies specifically focusing on these variables (the ’*p factor*’; Brislin et al., 2021). This factor, following Caspi and collaborators (2014) could represent a person’s vulnerability to mental disorders and their comorbidity. Finally, the ‘extracurricular activities’ domain emerged as a small domain including the practice of sport and other extracurricular activities (e.g., music, art, hobbies). This is the less connected domain of the network, but it is a prominent aspect of children and adolescents lives because they freely choose and engage in such activities and their participation is important for different aspects of their lives, ranging from educational outcomes, to physical and mental health, skills, and well-being [108–111].

The five domains individuated are obviously not sufficient to entirely describe the environmental, social, and psychological aspects of children’s lives and key aspects are probably missing (e.g., personality traits, motivation, self-beliefs). Nonetheless, our model is still one of the most complete in literature—thanks to the large ABCD data collection—and we believe that the five domains well describe a large spectrum of children’s variability and that our results could well inform about the role of these domains for children’s positive affect, as discussed in the next paragraph.

### What makes children happy? Positive affect development and its predictors

Longitudinal studies about the development of positive affect between late childhood and early adolescence are not abundant, but, with some exceptions, they point to a decrease in happiness or positive affect during this period [22, 24]. Our results are in line with this finding as we show that positive affect decreases every 6 months (ca) by.06 points in *z* scores, which may lead to a decrease of almost half standard deviation in four years. This is an important point because it confirms that the decline of positive affect starts years before adolescence and, as researchers, we should be interested in finding ways to sustain children’s positive affect as it may lead to the successful or unsuccessful development of future health and wellbeing, psychopathology, and interpersonal relationships among the others [2, 10, 20, 32]. To this aim, we also studied the association between the five domains individuated by the EGA and positive affect. Indeed, starting from the literature it is difficult to discern which factors may have a major or minor role for positive affect in childhood and adolescence and all the five domains that we individuated could theoretically sustain children’s positive affect. In fact, there is plenty of research highlighting the role of—among the many—physical activity, environmental features, sleep, personality, interpersonal relationships, psychological problems, and maladaptive behaviors [33, 35, 36, 39–41, 44]. However, the different domains and the variables within and between them are often correlated, as evident for example in the relation between the characteristics of the social environment and impulsive or externalizing and internalizing behaviours [84, 112, 113]. For these reasons, we argued that a holistic approach to the question, that includes a high number of variables and their associations, could foster our understanding of the mechanisms underlying positive affect in children and adolescents.

Following this approach, we regressed the scores of the five domains on the latent intercept and the latent slope of positive affect. From these results, the primary role of a supportive social environment emerged. In fact, the ‘supportive social environment’ domain showed a medium-to-large positive association (*β* = .44) with the latent intercept of positive affect. Contrarily, none of the other factors showed a strong association with positive affect, as highlighted by standardized effect lower than |.10|, with the exception of ‘psychological problems’. In other words, practicing extracurricular activities, having higher cognitive abilities, lower psychological problems, or behaving less impulsively does not directly, or only slightly in the case of psychological problems, influence adolescents’ positive affect. The effect of a supportive social environment also resulted stable for all participants across time, as highlighted by the correlations in Table 2. This is particularly important given the instability of positive affect across time. Interestingly, correlation values between a ‘supportive social environment’ and positive affect are comparable to the mutual correlations between different observations of positive affect itself (see Table 2). This indicates that a supportive social environment, in the long run, could predict positive affect as well as baseline positive affect does.

These results are in line with the evidence in the literature about the great importance that the relationship with parents has for adolescents’ positive affect [32, 114–116], with adolescents also qualitatively reporting about the importance of their family relationship for their own well-being [117]. On the same line, quantitative and qualitative research strongly support the role that teachers and school environment play for students in middle and high school [118–122]. For example, a supportive environment at school and a good school climate are strong predictors of students’ mental health, positive relationships, academic outcomes, motivations, and emotions [122]. Our results, however, suggest that other factors might not be as effective in directly sustaining their happiness when they are included within a larger network of variables. This might have a great practical impact because, even if positive effects of psychological interventions aimed at increasing subjective well-being exist [123], institutions and organizations that aim at increasing adolescents happiness on a larger scale might shift (or keep) their focus to the social environments children are exposed to, instead of focusing on narrow aspects of their lives such as extracurricular activities. For example, school programs aimed at increasing students’ scholastic engagement, sense of belonging and school climate [121, 124–127] might be increasingly implemented with the additional aim, this time, of increasing adolescents’ happiness. This aligns with recent suggestions to prioritize school and classroom climate as predictors of students’ adjustment and wellbeing [127]. Social-emotional learning (SEL) programs, which aim to foster students’ skills to manage their emotions and establish and maintain supportive and caring relationships, might be well suited to this aim given their impact on students’ well-being, emotional competencies, schools’ climate, and attitude [128–131]. As also suggested by Oberle et al. [132], these results suggest strengthening and emphasizing strategies that may foster adolescents’ relationships with both peers and adults at school. Similarly, research and interventions might focus on parent trainings, trying to make them more effective and possibly focusing on promoting the relationships between parents and children in term of increasing children acceptance of their parents, and parents competences and self-esteem and lowering family conflicts [133–135]. In particular, it might be important to foster parents’ emotion socialization abilities, that might consequently help children to develop emotion regulation skills [136, 137]. Parents’ emotion regulation strategies and emotionally supportive responses are in fact associated with higher emotion regulation abilities and lower depressive symptoms in children [137]. These might also affect other aspects, such as adolescents’ behavioural and psychological problems [138]. In other words, investing in schools and families could be, again, a solution to promote children’s and adolescents’ happiness as well as other important factors, such as education, behaviours, or social relationships.

### Limitations and future directions

Although our study is based on a large and longitudinal data collection including more than 46 variables, it also has limitations that future studies could overcome. Firstly, our study is limited to a US population, which may restrict the generalizability of our findings. This is particularly pertinent for our main predictor, identified as the ’supportive social environment,’ which could exhibit markedly different characteristics in other contexts where family and school structures and values may vary. It is also important to note that we tested the longitudinal association between a set of baseline predictors and multiple measurements of positive affect. This follows a temporal relationship that we deemed causal, but cross-lagged panel models and multiple measurement of the predictors should be used to confirm the directionality of the relationships Future studies, once the ABCD data will be completely available, could tackle this question and unveil such important theoretical paths. The study is also limited to the variables available from the ABCD data collection, but other important factors (e.g., personality, social skills) could play a significant role for positive affect. However, following our study, future research could directly test the effects of such factors beyond that of a supportive social environment.

### Conclusions

Adopting a network approach applied to a very large sample size and using a longitudinal data collection, our study identified—for the first time—what are the main domains characterizing children’s cognitive, psychological and environmental measures. Also, not only confirmed that positive affect declines between 9 and 13 years old but, more importantly, deepened the fundamental question about “what makes children happy”. This allowed us to make reliable conclusions about the importance of specific domains—while taking into account the interconnectedness of many psychological variables—and to pinpoint what are the most important factors responsible for children’s and adolescents’ happiness: their families and schools, or, more in general, the presence of a supportive social environment. This is of primary importance for practitioners and policymakers as it might narrow their attention to what, within the complex network of our psychological, social, and environmental variability, effectively has a direct impact on positive affect.

## Supporting information

S1 Table List of the measures. (DOCX)

S2 Table Standardized loadings of the EGA model. (DOCX)

S1 Fig Stability of the nodes across replication. Numbers represent the percentage of times the node loaded on the expected domain.
(DOCX)
