# Supplementary material for: Happy children! A network of psychological and environmental factors associated with the development of positive affect in 9–13 children
Source: PLoS One. 2024 Sep 6;19(9):e0307560. doi: 10.1371/journal.pone.0307560 (PMC11379200; doi:10.1371/journal.pone.0307560)
Supplement: S1 Table — (DOCX) [file pone.0307560.s001.docx]

**Happy Children! A Network of Psychological and Environmental Factors Associated With the Development of Positive Affect in 9-13 children.**

# **Supplementary materials**

**Table S1.** List of the measures

| **Measure** | **Construct** | **Reference** |  |
| --- | --- | --- | --- |
| **Mental Health** | | | |
| UPPS-P Impulsive Behavior Scale | Lack of perseverance | Watts et al., 2020 |  |
|  | Lack of premeditation |  |  |
|  | Sensation seeking |  |  |
|  | Negative urgency |  |  |
|  | Positive urgency |  |  |
| Prodromal Psychosis Scale | Psychosis risk | Loewy et al., 2005 |  |
| BIS/BAS scale | Fun seeking | Pagliaccio et al., 2016 |  |
|  | Drive |  |  |
|  | Reward responsiveness |  |  |
|  | Behavioral inhibition |  |  |
| Friendship scale | Number of friends | ABCD |  |
| Parent General Behavior Inventory | Mania symptoms | Youngstrom et al., 2008 |  |
| Achenbach Child Behavior Check List | Behavioral problems | Achenbach, 2009 |  |
| Kiddie Schedule for Affective Disorders and Schizophrenia | Depression | (KSADS-5, Kaufman et al., 2021) |  |
|  | Anxiety disorders |  |  |
|  | Somatic disorders |  |  |
|  | ADHD |  |  |
|  | Conduct disorders |  |  |
|  | Oppositive behavior disorders |  |  |
|  | Obsessive compulsive disorder |  |  |
|  | Sluggish cognitive tempo |  |  |
|  | Stress disorder |  |  |
| **Physical Health** | | | |
| Youth Risk Behavior Survey | Weekly physical activity | CDC, 2016 |  |
| Screen Time Questionnaire for Child | Screen time | Hull et al., 2014; Sharif et al., 2010 |  |
| Sports and Activities Involvement Questionnaire | Sport participation | Huppertz et al., 2016 |  |
|  | Other activities (hobby) |  |  |
| Sleep Disturbance Scale for Children | Sleep disturbance | Ferreira et al., 2009 |  |
| **Environment** | | | |
| Prosocial Behavior Scale | Prosocial behavior | Goodman, 2001 |  |
| Parental monitoring | Parental monitoring | Karoly et al., 2016 |  |
| Child Report of Behavior Inventory | Parental warmth and acceptance | Schaefer, 1965; Schludermann & Schludermann, 1970 |  |
| Family Environment Scale | Family conflict | Moos & Moos, 1987 |  |
| School Risk and Protective Factors Survey | School environment | Arthur et al., 2007 |  |
|  | School involvement |  |  |
|  | School disengagement |  |  |
| ABCD Youth Neighborhood Safety/Crime Survey Modified from PhenX | Neighborhood safety | Echeverria et al., 2004 |  |
| **Neurocognition** | | | |
| Picture Sequence Memory Test | Episodic memory | <http://www.nihtoolbox.org> |  |
| Flanker Task | Inhibition and conflict monitoring |  |  |
| Dimensional Change Card Sort Task | Cognitive flexibility |  |  |
| List Sorting Working Memory Test | Working memory |  |  |
| Pattern Comparison Processing Speed Test | Processing speed |  |  |
| Picture Vocabulary Task | Verbal abilities |  |  |
| Oral Reading Recognition Task | Reading |  |  |
| Rey Auditory Verbal Learning Test | Learning, memory and recognition | Daniel et al., 2014 |  |
|  | Long term memory delay |  |  |
| Little Man Task | Visuospatial abilities | Acker & Acker, 1982 |  |
| Matrix Reasoning Task | Fluid intelligence | Wechsler, 2014 |  |
| **Positive Affects** | | | |
| Positive affects scale | Positive affects | Salsman et al., 2013 |  |
