# Supplementary material for: Happy children! A network of psychological and environmental factors associated with the development of positive affect in 9–13 children
Source: PLoS One. 2024 Sep 6;19(9):e0307560. doi: 10.1371/journal.pone.0307560 (PMC11379200; doi:10.1371/journal.pone.0307560)
Supplement: S2 Table — (DOCX) [file pone.0307560.s002.docx]

**Happy Children! A Network of Psychological and Environmental Factors Associated With the Development of Positive Affect in 9-13 children.**

# **Supplementary materials**

**Table S2.** Standardized loadings of the EGA model

| Node | Construct | Standardized loadings | | | | |
| --- | --- | --- | --- | --- | --- | --- |
|  |  | D1 | D2 | D3 | D4 | D5 |
| V12 | Prodromal psychosis | .39 |  |  |  |  |
| V13 | Negative urgency | .51 |  |  |  |  |
| V15 | Sensation seeking | .28 |  |  |  |  |
| V16 | Positive urgency | .55 |  |  |  |  |
| V18 | Behavioral inhibition (bis) | .38 |  |  |  |  |
| V19 | Reward responsiveness (bas) | .51 |  |  |  |  |
| V20 | Drive (bas) | .58 |  |  |  |  |
| V21 | Fun seeking (bas) | .62 |  |  |  |  |
| V22 | Friends | .14 |  |  |  |  |
| V34 | Weekly physical activity | -.02 |  |  |  |  |
| V35 | Screen time | .34 |  |  |  |  |
| V42 | Family conflict | .32 |  |  |  |  |
| V46 | Safe neighbourhood | -.19 |  |  |  |  |
| V1 | Vocabulary |  | .60 |  |  |  |
| V2 | Inhibition |  | .46 |  |  |  |
| V3 | Working memory |  | .62 |  |  |  |
| V4 | Cognitive flexibility |  | .54 |  |  |  |
| V5 | Processing speed |  | .38 |  |  |  |
| V6 | Episodic memory |  | .52 |  |  |  |
| V7 | Reading |  | .61 |  |  |  |
| V8 | Verbal memory |  | .65 |  |  |  |
| V9 | Fluid reasoning |  | .58 |  |  |  |
| V10 | Long term memory |  | .66 |  |  |  |
| V11 | Visuospatial abilities |  | .47 |  |  |  |
| V23 | Symptoms of mania |  |  | .64 |  |  |
| V24 | Total ext-int problems |  |  | 1.00 |  |  |
| V25 | Depression |  |  | .78 |  |  |
| V26 | Anxiety disorders |  |  | .73 |  |  |
| V27 | Somatic disorder |  |  | .48 |  |  |
| V28 | ADHD |  |  | .79 |  |  |
| V29 | Oppositional conductive disorders |  |  | .77 |  |  |
| V30 | Conduct disorders |  |  | .74 |  |  |
| V31 | Sluggish cognitive tempo |  |  | .61 |  |  |
| V32 | Obsessive compulsive disorders |  |  | .72 |  |  |
| V33 | Stress disorders |  |  | .90 |  |  |
| V38 | Sleep disturbance |  |  | .57 |  |  |
| V14 | Lack of planning |  |  |  | .51 |  |
| V17 | Lack of perseverance |  |  |  | .53 |  |
| V39 | Prosocial behaviour |  |  |  | -.53 |  |
| V40 | Parental monitoring |  |  |  | -.47 |  |
| V41 | Parent’s acceptance |  |  |  | -.47 |  |
| V43 | School environment |  |  |  | -.65 |  |
| V44 | School involvement |  |  |  | -.76 |  |
| V45 | School disengagement |  |  |  | .54 |  |
| V36 | Sport activity |  |  |  |  | .54 |
| V37 | Hobbies/other activities |  |  |  |  | .40 |

*Note.* D1 = behavioural dysregulation; D2 = cognitive functioning; D3 = psychological problems; D4 = supportive social environment; D5 = extracurricular activities
