# Supplementary material for: Happy children! A network of psychological and environmental factors associated with the development of positive affect in 9–13 children
Source: PLoS One. 2024 Sep 6;19(9):e0307560. doi: 10.1371/journal.pone.0307560 (PMC11379200; doi:10.1371/journal.pone.0307560)
Supplement: S1 Fig — Numbers represent the percentage of times the node loaded on the expected domain. (DOCX) [file pone.0307560.s003.docx]

**Happy Children! A Network of Psychological and Environmental Factors Associated With the Development of Positive Affect in 9-13 children.**

# **Supplementary materials**

**Figure S1.** Stability of the nodes across replication. Numbers represent the percentage of times the node loaded on the expected domain.


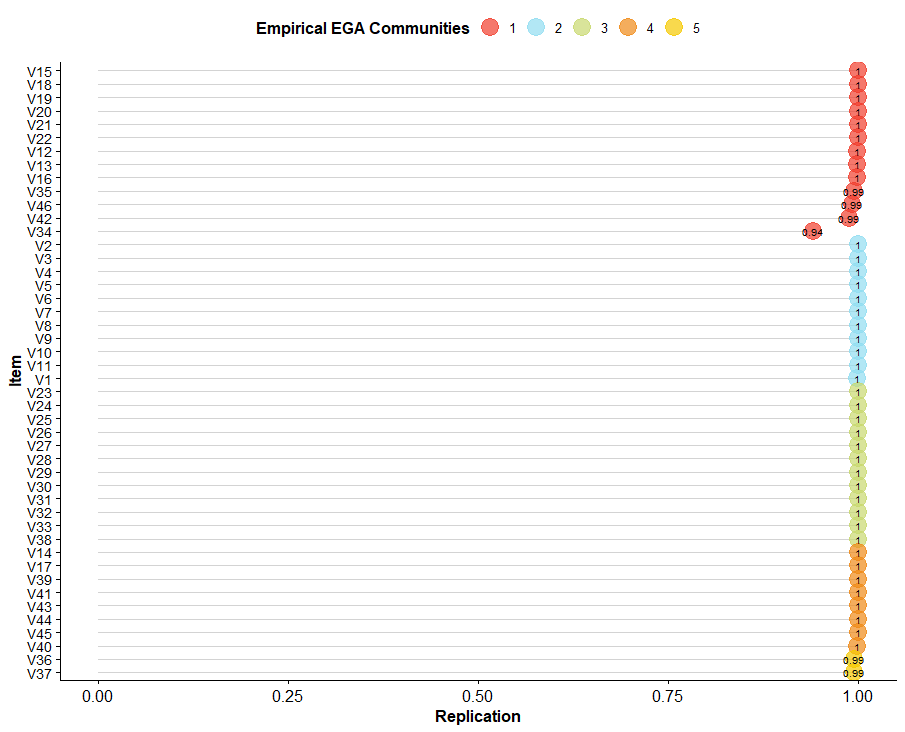


| V1= vocabulary | V17= Lack of perseverance | V33= stress disorders |
| --- | --- | --- |
| V2= inhibition | V18= behavioral inhibition (bis) | V34= weekly physical activity |
| V3= working memory | V19= reward responsiveness (bas) | V35= screen time |
| V4= cognitive flexibility | V20= drive (bas) | V36= sport activity |
| V5= processing speed | V21= fun seeking (bas) | V37= hobbies/other activities |
| V6= episodic memory | V22= friends | V38= sleep disturbance |
| V7= reading | V23= symptoms of mania | V39= prosocial behaviour |
| V8= verbal memory | V24= total ext-int problems | V40= parental monitoring |
| V9= fluid reasoning | V25= depression | V41= parent’s acceptance |
| V10= long term memory | V26= anxiety disorders | V42= family conflict |
| V11= visuospatial abilities | V27= somatic disorder | V43= school environment |
| V12= Prodromal psychosis | V28= ADHD | V44= school involvement |
| V13= negative urgency | V29= oppositional conductive dis | V45=school disengagement |
| V14= lack of planning | V30= conduct disorders | V46= safe neighbourhood |
| V15= sensation seeking | V31= sluggish cognitive tempo |  |
| V16= positive urgency | V32= obsessive compulsive disorders |  |
